# Supplementary material for: Using microbiological data to improve the use of antibiotics for respiratory tract infections: A protocol for an individual patient data meta-analysis
Source: PLoS One. 2023 Nov 27;18(11):e0294845. doi: 10.1371/journal.pone.0294845 (PMC10681295; doi:10.1371/journal.pone.0294845)
Supplement: S1 Appendix — (DOCX) [file pone.0294845.s002.docx]

**Appendix one: search terms**

| **#** | **Query** | **Results from 14 Nov 2022** |
| --- | --- | --- |
| 1 | respiratory tract infection/ or influenza/ or laryngotracheobronchitis/ or lower respiratory tract infection/ or respiratory syncytial virus infection/ or upper respiratory tract infection/ or viral respiratory tract infection/ | 102,631 |
| 2 | respiratory tract infections.mp. | 48,812 |
| 3 | upper respiratory tract infection/ or viral upper respiratory tract infection/ | 42,020 |
| 4 | upper respiratory tract infection.mp. | 3,380 |
| 5 | laryngitis.mp. | 4,013 |
| 6 | pharyngitis/ | 8,620 |
| 7 | pharyngitis.mp. | 10,917 |
| 8 | common cold/ or rhinitis/ or rhinovirus infection/ | 18,566 |
| 9 | common cold.mp. | 6,247 |
| 10 | rhinitis.mp. | 46,239 |
| 11 | lower respiratory tract infection.mp. | 3,096 |
| 12 | sinusitis/ or acute sinusitis/ or bacterial sinusitis/ or chronic sinusitis/ or frontal sinusitis/ or rhinosinusitis/ or viral sinusitis/ | 20,245 |
| 13 | sinusitis.mp. | 28,010 |
| 14 | tracheitis/ or tracheobronchitis/ | 1,555 |
| 15 | tracheitis.mp. | 1,907 |
| 16 | pneumococcal disease.mp. | 3,957 |
| 17 | cough.mp. | 56,596 |
| 18 | sneez*.mp. | 4,121 |
| 19 | sneezing/ | 979 |
| 20 | influenza/ | 56,157 |
| 21 | influenza.mp. | 115,539 |
| 22 | otitis media/ | 18,043 |
| 23 | otitis media.mp. | 29,273 |
| 24 | dyspnea/ | 24,112 |
| 25 | breatheless*.mp. | 0 |
| 26 | fever/ | 42,573 |
| 27 | fever.mp. | 218,919 |
| 28 | rhinorrhea/ | 73 |
| 29 | runny nose.mp. | 752 |
| 30 | sore throat/ | 8,620 |
| 31 | sore throat.mp. | 5,393 |
| 32 | wheezing.mp. | 9,373 |
| 33 | wheezing/ or abnormal respiratory sound/ | 9,860 |
| 34 | bacterial infection/ | 74,211 |
| 35 | bacter*.mp. | 1,597,454 |
| 36 | respiratory synctial virus.mp. | 20 |
| 37 | virus pneumonia/ or Respiratory syncytial pneumovirus/ or virus infection/ | 41,226 |
| 38 | Adenoviridae/ | 28,625 |
| 39 | adenovirus.mp. | 44,376 |
| 40 | haemophilus influenzae/ or haemophilus influenzae type a/ or haemophilus influenzae type b/ | 14,227 |
| 41 | haemophilus influenzae.mp. | 21,621 |
| 42 | Staphylococcus aureus/ | 70,984 |
| 43 | staphylococcus aureus.mp. | 125,949 |
| 44 | Moraxella catarrhalis/ | 2,038 |
| 45 | Moraxella catarrhalis.mp. | 3,311 |
| 46 | Streptococcus pneumoniae/ | 24,335 |
| 47 | Streptococcus pneumoniae.mp. | 34,028 |
| 48 | Mycoplasma pneumoniae/ | 3,524 |
| 49 | Mycoplasma pneumoniae.mp. | 6,490 |
| 50 | Chlamydia pneumoniae/ | 3,840 |
| 51 | Chlamydia pneumoniae.mp. | 3,816 |
| 52 | influenza a virus/ | 22,546 |
| 53 | Influenza virus a.mp. | 2,332 |
| 54 | Influenza virus b.mp. | 189 |
| 55 | Parainfluenza virus.mp. | 6,020 |
| 56 | Bordetella pertussis/ | 5,588 |
| 57 | Bordetella pertusis.mp. | 6 |
| 58 | Bordetella parapertusis.mp. | 2 |
| 59 | human metapneumovirus/ | 1,465 |
| 60 | Human metapneumovirus.mp. | 1,786 |
| 61 | Beta haemolytic streptococc.mp. | 0 |
| 62 | metapneumovirus/ | 1,465 |
| 63 | Parechovirus.mp. or Human parechovirus/ or Parechovirus/ or human parechovirus test kit/ or parechovirus infection/ | 671 |
| 64 | Bocavirus.mp. | 1,160 |
| 65 | bronchiolitis/ or viral bronchiolitis/ | 5,527 |
| 66 | bronchitis/ or chronic bronchitis/ | 21,899 |
| 67 | laryngitis/ | 3,231 |
| 68 | Clinical trial/ | 535,652 |
| 69 | Randomized controlled trial/ | 579,313 |
| 70 | Randomization/ | 106,890 |
| 71 | Single blind procedure/ | 0 |
| 72 | Double blind procedure/ | 0 |
| 73 | Crossover procedure/ | 0 |
| 74 | Placebo/ | 0 |
| 75 | Randomi?ed controlled trial$.tw. | 197,756 |
| 76 | Rct.tw. | 24,010 |
| 77 | Random allocation.tw. | 1,666 |
| 78 | Randomly allocated.tw. | 29,323 |
| 79 | Allocated randomly.tw. | 2,215 |
| 80 | (allocated adj2 random).tw. | 772 |
| 81 | Single blind$.tw. | 19,350 |
| 82 | Double blind$.tw. | 152,565 |
| 83 | ((treble or triple) adj blind$).tw. | 1,059 |
| 84 | Placebo$.tw. | 220,000 |
| 85 | controlled study.mp. or controlled study/ | 45,610 |
| 86 | controlled clinical trial.mp. or controlled clinical trial/ | 109,783 |
| 87 | Family study/ | 0 |
| 88 | Longitudinal study/ | 161,060 |
| 89 | Retrospective study/ | 1,069,146 |
| 90 | Prospective study/ | 641,988 |
| 91 | Cohort analysis/ | 320,674 |
| 92 | (Cohort adj (study or studies)).mp. | 464,466 |
| 93 | (follow up adj (study or studies)).tw. | 50,297 |
| 94 | (observational adj (study or studies)).tw. | 123,139 |
| 95 | (epidemiologic$ adj (study or studies)).tw. | 84,457 |
| 96 | (primary care or primary health or family physician* or general practi* or family practi* or outpatient? or clinic? or ambulatory or health centre? or health centre? or office).ti,ab. | 784,460 |
| 97 | 1 or 2 or 3 or 4 or 5 or 6 or 7 or 8 or 9 or 10 or 11 or 12 or 13 or 14 or 15 or 16 or 17 or 18 or 19 or 20 or 21 or 22 or 23 or 24 or 25 or 26 or 27 or 28 or 29 or 30 or 31 or 32 or 33 or 65 or 66 or 67 | 574,281 |
| 98 | 34 or 35 or 36 or 37 or 38 or 39 or 40 or 41 or 42 or 43 or 44 or 45 or 46 or 47 or 48 or 49 or 50 or 51 or 52 or 53 or 54 or 55 or 56 or 57 or 58 or 59 or 60 or 61 or 62 or 63 or 64 | 1,754,568 |
| 99 | 68 or 69 or 70 or 71 or 72 or 73 or 74 or 75 or 76 or 77 or 78 or 79 or 80 or 81 or 82 or 83 or 84 or 85 or 86 or 87 or 88 or 89 or 90 or 91 or 92 or 93 or 94 or 95 | 3,186,812 |
| 100 | 96 and 97 and 98 and 99 | 2,610 |
| 101 | limit 100 to yr="2008 - 2022" | 1,496 |
